# Identification of protein clusters predictive of tumor response in rectal cancer patients receiving neoadjuvant chemo-radiotherapy

**DOI:** 10.18632/oncotarget.16053

**Published:** 2017-03-09

**Authors:** Ombretta Repetto, Valli De Re, Antonino De Paoli, Claudio Belluco, Lara Alessandrini, Vincenzo Canzonieri, Renato Cannizzaro

**Affiliations:** ^1^ Facility of Bio-Proteomics, Immunopathology and Cancer Biomarkers, IRCCS CRO National Cancer Institute, Aviano, Italy; ^2^ Radiation Oncology, IRCCS CRO National Cancer Institute, Aviano, Italy; ^3^ Surgical Oncology, IRCCS CRO National Cancer Institute, Aviano, Italy; ^4^ Pathology, IRCCS CRO National Cancer Institute, Aviano, Italy; ^5^ Gastroenterology, IRCCS CRO National Cancer Institute, Aviano, Italy

**Keywords:** DIGE, gastric diseases, rectal proteomics, rectal cancer, tumor regression grade

## Abstract

Preoperative neoadjuvant chemoradiotherapy (nCRT) is the gold standard in locally advanced rectal cancer, only 10–30% of patients achieving benefits. Currently, there is a need of a reliable selection of markers for the identification of poor or non-responders prior to therapy. In this work, we compared protein profiles before therapy of patients differing in their responses to nCRT to find novel predictive markers of response to therapy. Patients were grouped into 3 groups according to their tumor regression grading (TRG) after surgery: 'TRG 1–2′, good responders, 'TRG 3′ and 'TRG 4′, poor responders. Paired surgical specimens of rectal cancer and healthy (histologically confirmed) rectal tissues from 15 patients were analysed before nCRT by two dimensional difference in gel electrophoresis followed by mass spectrometry. Thirty spots were found as differentially expressed (*p* < 0.05). Among them, 3 spots (spots 471, 683 and 684) showed an increased amount of protein in poor responders compared with good responders, and they were more tumor associated compared with healthy tissues. Proteins of these spots were identified as fibrinogen ß chain fragment D, actin isoforms, B9 and B5 serpins, cathepsin D isoforms and peroxiredoxin-4. In an independent validation set of 20 rectal carcinomas we validated the increased fibrinogen ß chain abundance before nCRT in poor responders by immunoblotting. In conclusion, we propose a risk-stratification tool in predicting the response to nCRT treatment in rectal cancer based on the quantity of these proteins.

## INTRODUCTION

Rectal cancer (RC) comprises about 2.4% of all human malignancies [1]. In locally advanced rectal cancer (LARC), the standard of care is surgical resection preceded by neoadjuvant chemoradiotherapy (nCRT). A complete response occurs in approximately 10–30% of patients [2]. Complex molecular and clinical phenotypes underlie the development and progression of rectal carcinoma, thus giving rise to the high variability in term of tumor responses to treatment [3].

At present, tumor regression grading (TRG) is one of the most common criteria to evaluate tumor responses to nCRT, and it also possesses a potential value as an independent prognostic factor for disease-free survival and patient's outcome [4–8].

In the last years, there has been an intense interest in the individuation of molecular pathways in LARC, in order to find out predictive markers of response to CRT and to spare non-responsive patients from unnecessary treatment [9–11]. For these reasons, different approaches (e.g. microarray, single nucleotide polymorphism, DNA methylation, immunohistochemistry) have been adopted to investigate potential changes in genes involved in crucial pathways (e.g. angiogenesis, cell proliferation drug delivery, DNA repair) and to characterize common signatures distinguishing patients poor or not-responders prior to therapy [11–16].

At protein level, first attempts for serum or tumor tissue biomarker discovery identified a set of proteins, peptides and phosphorylation levels discriminating between responders and nonresponders before therapy [17–20]. In spite of the consistent amount of studies, none of the proposed biomarkers have sufficient evidence to support their use in clinical practice.

In this work, we present the results of two dimensional differential in gel electrophoresis (2D-DIGE) proteomic study performed on RC biopsies. Subgroups analysis was performed based on prognostic value of TRG for tumor nCRT response, in an effort to discover biomarkers predictive of nCRT response and to accomplish an optimal therapy for patients with RC.

## RESULTS

### 2D-DIGE differential spots associated with good (TRG 1-2) or poor responses (TRG 3 and TRG4)

All patients included in this study underwent the full course of nCRT followed by surgical excision of the tumor. Demografics details and staging informations are showed in Table 1 and Supplementary Table 1.

**Table 1:** Clinicopathological characteristics of patients affected by rectal cancer of median and distal localization, accordantly to the criteria for the nCRT [21]

Patient nr.	Sex	Age	Pre-CRT stage TNM	TRG	TRG group of analysis
1	F	62	T3N0	1	1-2
2	F	51	T3N+	2	
3	M	62	T1/2N0	1	
4	M	59	T3N+	1	
5	M	64	T3N+	3	3
6	M	66	T3N0	3	
7	M	49	T3N+	3	
8	M	59	T3N+	3	
9	M	68	T3N0	3	
10	M	58	T3N+	3	
11	M	43	T3N+	3	
12	F	64	T3/4N+	4	4
13	M	61	T3N0	4	
14	M	83	T3N0	4	
15	M	67	T3N0	4	

Pathological responses were evaluated with the tumor regression grade (TRG) system.

**Figure 1:**
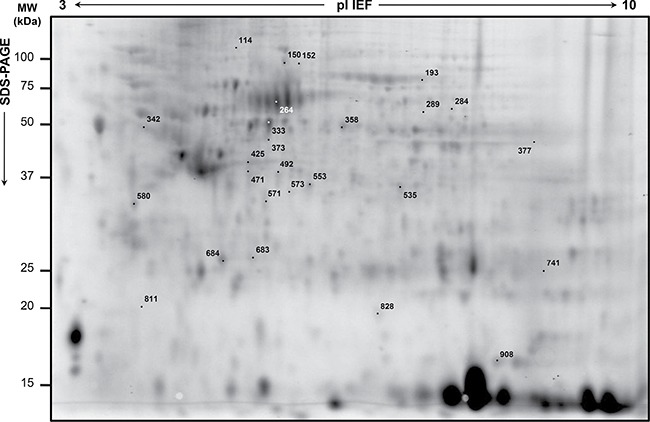
Representative analytical proteome map of rectal cancer (RC) Proteins were resolved by isoelectrofocusing over the pI 3-10, followed by 8–16% gradient second dimension. Numbered spots indicate the differentially expressed spots in RC biopsies of ‘TRG 1-2′ *versus* either ‘TRG 3′ or ‘TRG 4′. Identified proteins are listed in Table 2.

The proteomic workflow adopted is illustrated in Supplementary Figure 1. The 2D-DIGE differential patterns were obtained from matches among RC biopsies collected before preoperative nCRT from 15 patients with good (‘TRG 1–2′), moderate (‘TRG 3′) or partial (‘TRG 4′) responses to nCRT. A total of 30 spots significantly varied (*p* < 0.05) in content between ‘TRG 1–2′ patients *versus* ‘TRG 3′ or ‘TRG 4′ ones (Figure 1; Table 2). In particular, 16 spots were differentially expressed in ‘TRG 1–2′ *versus* ‘TRG 3′, and 14 spots resulted differentially expressed in ‘TRG 1–2′ *versus* ‘TRG 4′. Four spots (377, 471, 683 and 684) increased in content in both ‘TRG 3′ and ‘TRG 4′ *versus* ‘TRG 1–2′.

**Table 2:** Differentially expressed proteins of ‘TRG 1-2′ group related to rectal cancer (RC) in comparison with those of either ‘TRG 3′ or ‘TRG 4′ groups

Spot nr.^a^)	MW (Da)/pI	Accession	Gene	Protein annotation	Cellular locationb)	Fold Δ	*p*-value
**Class a) Up-regulated spots in ‘TRG 1-2′ *versus* ‘TRG 3′ (nr = 5)**							
114	99551/5.34	MVP_HUMAN	MVP	**Major vault protein**	nucleus, nuclear pore complex	2.2	0.023
150	84026/6.08	IMMT_HUMAN	IMMT	**Mitochondrial inner membrane protein or mitofilin**	mitochondrion inner membrane	1.7	0.048
152	82973/6.15	gi|154354966	IMMT	**Mitochondrial inner membrane protein isoform 3 or mitofilin**	mitochondrion inner membrane	1.6	0.012
333	54541/5.61	gi|220702506	TAPBP, PDIA3	**Chain A, TapasinERP57 HETERODIMER**	endoplasmic reticulum	1.5	0.015
358	57794/6.01	TCPB_HUMAN	CCT2	**T-complex protein 1 subunit ß**	cytoplasm	1.5	0.011
**Class b) Up-regulated spots in ‘TRG 1-2′ *versus* ‘TRG 4′ (nr = 3)**							
908	18229/7.68	PPIA_HUMAN	PPIA	**Peptidyl-prolyl cis-trans isomerase or Cyclophilin A**	cytoplasm, secreted	2.8	0.046
289	79805/7.7	gi|34228	LMNA	**Unnamed protein product, putative lamin A precursor**		1.8	0.010
571	36088/5.84	gi|1703319	ANXA4	**Annexin A4**	cytoplasm, cell surface, membranes, nucleus, secreted	1.7	0.010
**Class c) Up-regulated spots in ‘TRG 3′ *versus* ‘TRG 1-2′ (nr = 11)**							
373	53548/6	gi|148491091	SLC25A24	**Calcium-binding mitochondrial carrier protein SCaMC-1 isoform 1**	mitochondrion inner membrane	−1.5	0.028
535	36892/6.32	AK1A1_HUMAN	ADH1A	**Alcohol dehydrogenase [NADP(+)]**	cytoplasm, apical plasma membrane, extracellular exosome, extracellular space	−1.5	0.035
811	19871/4.80	MYL9_HUMAN	MYL9	**Myosin regulatory light polypeptide 9**	cytoplasm, muscle myosin, stress fiber, Z disc	−1.5	0.019
377	59828/9.16	ATPA_HUMAN	ATP5A1	**ATP synthase subunit α, mitochondrial**	mitochondrion inner membrane, cell membrane, peripheral membrane protein, extracellular side	−1.6	0.044
471	56577/8.54	FIBB_HUMAN	FGB	**Fibrinogen ß chain**	secreted	−1.6	0.022
	42052/5.29	ACTB_HUMAN	ACTB	**Actin, cytoplasmic 1**	cytoplasm, cytoskeleton		
	43004/5.61	SPB9_HUMAN	SERPINB9	**Serpin B9**	cytoplasm		
	42530/5.72	SPB5_HUMAN	SERPINB5	**Serpin B5**	cytoplasm		
580	32856/4.72	gi|63252900	TPM1	**Tropomyosin α-1 chain isoform 4**	cytoplasm, cytoskeleton	−1.9	0.013
284	59947/6.90	CATA_HUMAN	CAT	**Catalase**	peroxisome	−2.1	0.016
573	37688	TALDO_HUMAN	TALDO1	**Transaldolase**	cytoplasm	−2.1	0.005
492	56577/8.54	FIBB_HUMAN	FGB	**Fibrinogen ß chain**	secreted	−2.2	0.002
	42052/5.29	ACTB_HUMAN	ACTB	**Actin, cytoplasmic 1**	cytoplasm, cytoskeleton		
	42540/5.72	gi|60817455	SERPINB5	**Serpin B5**	cytoplasm		
683	42052/5.29	ACTB_HUMAN	ACTB	**Actin, cytoplasmic 1**	cytoplasm, cytoskeleton	−2.3	0.003
	30749/5.86	PRDX4_HUMAN	PRDX4	**Peroxiredoxin-4**	cytoplasm, secreted		
	45037/6.10	CATD_HUMAN	CTSD	**Cathepsin D**	secreted, extracellular space		
684	42052/5.29	ACTB_HUMAN	ACTB	**Actin, cytoplasmic 1**	cytoplasm, cytoskeleton	−2.8	0.003
	45037/6.10	CATD_HUMAN	CTSD	**Cathepsin D**	secreted, extracellular space		
**Class d) Up-regulated spots in ‘TRG 4′ *versus* ‘TRG 1-2′ (*n* = 11)**							
193	74380/6.57	LMNA_HUMAN	LMNA	**Prelamin-A/C**	nucleus (nucleoplasm, lamina, envelope)	−1.3	0.030
	79294/6.81	TRFE_HUMAN	TF	**Serotransferrin**	secreted		
342	56525/5.26	ATPB_HUMAN	ATP5B	**ATP synthase subunit ß, mitochondrial**	mitochondrion inner membrane, cell membrane, peripheral membrane protein	−1.6	0.021
	50095/4.78	TBB5_HUMAN	TUBB	**Tubulin ß chain**	cytoplasm		
264	68988/5.69	gi|157830361	ALB	**Chain A, Human Serum Albumin In A Complex With Myristic Acid And Tri- Iodobenzoic Acid**	extracellular space	−1.7	0.025
471	56577/8.54	FIBB_HUMAN	FGB	**Fibrinogen ß chain**	secreted	−1.9	0.009
	42052/5.29	ACTB_HUMAN	ACTB	**Actin, cytoplasmic 1**	cytoplasm, cytoskeleton		
	43004/5.61	SPB9_HUMAN	SERPINB9	**Serpin B9**	cytoplasm		
683	42052/5.29	ACTB_HUMAN	ACTB	**Actin, cytoplasmic 1**	cytoplasm, cytoskeleton	−1.9	0.040
	30749/5.86	PRDX4_HUMAN	PRDX4	**Peroxiredoxin-4**	cytoplasm, secreted		
	45037/6.10	CATD_HUMAN	CTSD	**Cathepsin D**	secreted, extracellular space		
425	42215/5.91	ACTBM_HUMAN	POTEI	**Putative ß-actin-like protein 3 (POTE ankyrin domain family member K)**	cytoplasm, cytoskeleton	−2.3	0.019
741	23883/5.35	gi|194173391		**Immunoglobulin light chain**	secreted	−2.4	0.043
553	34726/6.06	PSDE_HUMAN	PSMD14	**26S proteasome non-ATPase regulatory subunit 14**	cytoplasm, proteasome	−2.5	0.035
684	42052/5.29	ACTB_HUMAN	ACTB	**Actin, cytoplasmic 1**	cytoplasm, cytoskeleton	−2.5	0.031
	45037/6.10	CATD_HUMAN	CTSD	**Cathepsin D**	secreted, extracellular space		
377	59828/9.16	ATPA_HUMAN	ATP5A1	**ATP synthase subunit α, mitochondrial**	mitochondrion inner membrane, cell membrane, peripheral membrane protein, extracellular side	−2.7	0.008
828	20876/8.69	gi|62897565	TAGLN	**Transgelin variant**	cytoplasm	−3.8	0.050

^a^)spot nr., spot numbers refer to Figure 2; ^b^)keywords of cellular location from UniProtKB (http://www.uniprot.org/uniprot/) are reported.

**Figure 2:**
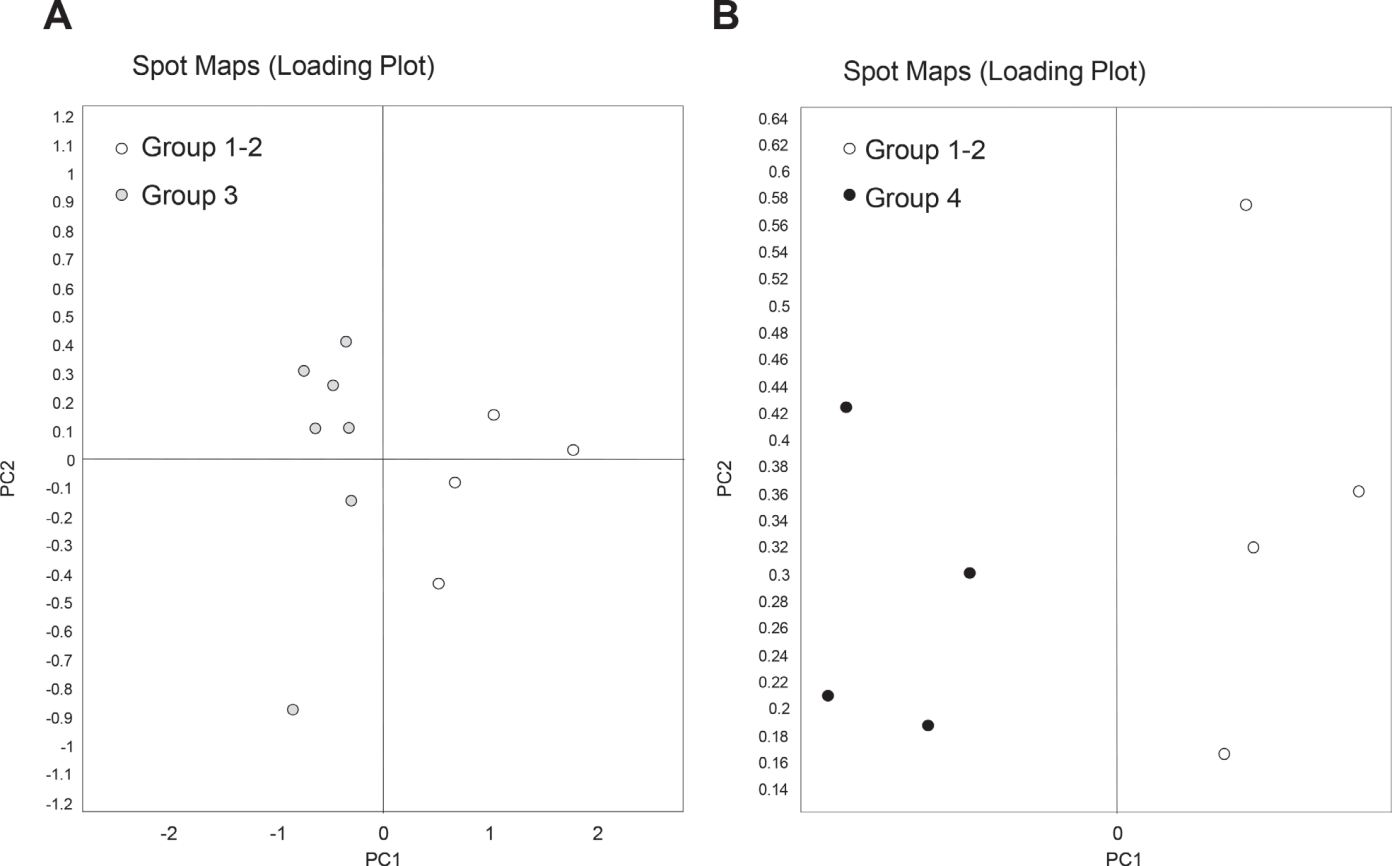
Principal component analysis of rectal cancer (RC) biopsies belonging to good responders (TRG 1-2) and poor responders (TRG3 and TRG4) Loading plots show an overview of the all spot maps from all groups. Proteome maps of ‘TRG 1–2′ have been compared with those of either ‘TRG 3′ (**A**) or ‘TRG 4′ (**B**). Each circle represents a spot map of a surgical specimen collected from one patient.

Principal component analysis showed that protein maps of cancer tissues belonging to good and poor responders were well separated, consistently with the occurrence of differential protein patterns (Figure 2).

### Protein identification from protein spots

Proteins from the 30 differentially expressed spots were successfully identified (Table 2; Supplementary Table 2). Spot 425, whose putative identity was a β-actin-like protein, was considered not reliable because of the low sequence coverage (3%) and the low number of peptides < 2. Spot 289 was identified as an ‘unnamed protein product’ with homology to a ‘lamin isoform A-delta50′ (NCBInr accession number: P_001269555). In the spots 193, 342, 471, 492, 683 and 684, more than one protein was identified. In other cases, a same protein was detected from different spots (i.e. ‘mitochondrial inner membrane protein’ in spots 150 and 152; ‘fibrinogen ß chain’ in spots 471 and 492; ‘actin’ in spots 471, 492, 683 and 684; ‘cathepsin D’ in spots 683 and 684), this may be coming from protein post-translational modifications or proteolytic cleavages. Possible phosphorylation events in STY aminoacids were not evidenced in proteins identified in spots 471, 492, 683 and 684. A total of 27 unique protein identifications were achieved. They were mostly located in the cytoplasm or secreted, with the exception of some proteins located in mitochondria (spots 150, 152, 373, 377 and 342), peroxisomes (spot 284) or nuclei (spots 114 and 193). Some proteins had also a possible multiple cellular location.

### 2D-DIGE spots and proteins with altered intensity in the tumor samples compared with their normal counterpart

**Figure 3:**
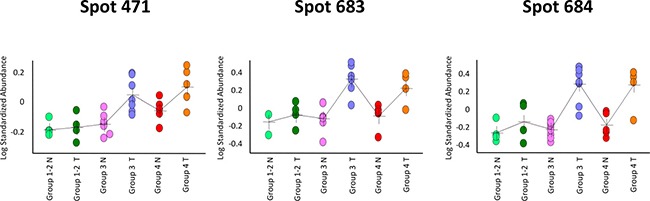
Graphical visualization abundance distribution of spots 471, 683 and 684 in rectal tumor (T) and healthy normal tissue (N) tissues The three differential spots increased in content in poor responders (‘TRG 3′ and ‘TRG 4′) *versus* good responders (‘TRG 1-2′), and had a higher content in cancer tissues than the healthy normal ones. In each graph, a single circle represents the Log standardized abundance of the spot calculated for one gel/patient.

For all the 30 differential spots and for each paired surgical specimen analysed by 2D-DIGE, spot content in tumor tissue (T) was compared with that in the healthy normal tissue (N). Data about the average ratio of spot abundance in N *versus* T tissues are reported in Table 3, where *p* < 0.05 values are indicated. A cancer specific localization was found for spots 471, 683 and 684, which increased in content in cancer tissues of the poor responsive patients of ‘TRG 3′ and ‘TRG 4′ groups. Figure 3 graphically shows their Log standardize abundance in N and T biopsies of the three TRG groups of patients.

**Table 3:** Difference in content of the 30 differential spots found in rectal cancer proteomes before nCRT

Class	Spot nr. ^a^)	Group TRG 1-2	Group TRG 3	Group TRG 4
**Up-regulated spots in TRG 1-2 versus either TRG 3 or TRG 4**				
	114	−1.21	−1.68	−1.56
	150	1.12	1.92 (*2.9E–005*)	1.05
	152	1.12	1.39 (*0.0057*)	1.20
	333	−1.12	1.30 (*0.016*)	1.31
	358	−1.32 (*0.05*)	1.06	1.28
	908	−2.24	−1.09	−1.14
	289	1	0.70	0.19
	571	−1.62	−1.05	−1.23
**Down-regulated spots in TRG 1-2 versus either TRG 3 or TRG 4**				
	373	1.07	−1.59 (*6.1E–005*)	−1.44
	535	1.20	−1.11	−1.03
	811	−1.29	−1.56 (*0.0051*)	−1.43
	377	2.18 (*0.03*)	1.33	−1.43
	471	−1.04	−1.59 (*0.0013*)	−1.47 (*0.04*)
	580	−1.30	−2.29 (*2.0E–005*)	−2.02
	284	1.32	−1.40 (*0.0084*)	−1.40
	573	1.51 (*0.04*)	−1.43 (*0.0085*)	−1.09
	492	−2.00	−3.22 (*7.0E–005*)	−2.13
	683	−1.20	−2.47 (*8.6E–005*)	−1.96 (*0.02*)
	684	−1.45	−3.41 (*7.0E–006*)	−2.94 (*0.005*)
	193	1.39 (*0.03*)	1.36 (*0.004*)	1.12
	342	1.12	1.04	−1.28 (*0.03*)
	264	1.41 (*0.072*)	1.67 (*0.0021*)	−1.08
	425	1.03	1.02	−1.48
	741	n.d.^b)^	1.37	−1.81
	553	−1.01	−1.84	−1.88
	828	1.52	−1.18	−2.72

^a)^spot nr., spot numbers refer to Figure 1; ^b^)n.d., not detected.

Within each TRG group, spot content has been compared in normal (N) versus tumor (T) biopsies, and its variation in abundance has been expressed as ‘average ratio’, where values > 1 refer to an increase in N, while values < −1 refer to an increase in T. The reported *p*-values refer to Student's *t* test < 0 .05.

### Immunoblotting and validation of fibrinogen β chain fragment D increase in poor responders

**Figure 4:**
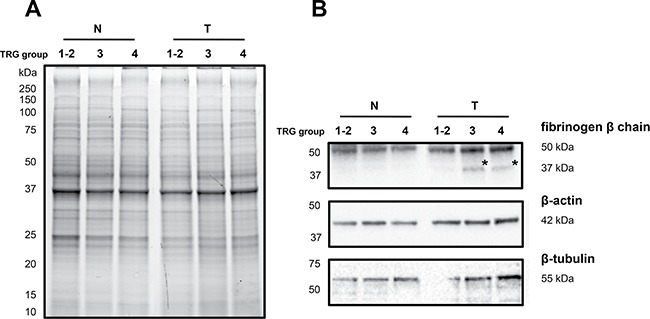
Immunoblotting analyses of three differentially expressed proteins in healthy normal (N) *versus* rectal tumor (T) tissues belonging to good responders (TRG 1-2) and poor responders (TRG3 and TRG4) (**A**) Image of the 1DE gel acquired with Chemidoc before its transfer to nitrocellulose membrane. (**B**) Signals of proteins cross-reacting with antibodies directed against fibrinogen β-chain, β-actin and β-tubulin. Asterisk indicates the signal of a cross-reacting band at around 37 kDa.

Firstly, immunoblotting using antibody against fibrinogen β chain was performed on pooled protein extracts used in 2D-DIGE analysis. One-dimensional electrophoresis revealed that the amount of total protein loading among samples was homogeneous (Figure 4A). As visualized by immunoblotting, content of fibrinogen β chain as band of ~50 kDa, corresponding to the weight of the protein, was higher in T samples than in N ones (Figure 4B). A band of ~37 kDa was detected in the only ‘TRG 3′ and ‘TRG 4′ T samples. This molecular weight of ~37 kDa was the same as the 2D-DIGE differential spot 471, which was identified as fibrinogen β chain by mass spectrometry in the protein portion between aminoacid positions 164 and 491, also known as fragment D (gi2781208). No significantly differential spot at the MW of around 50 kDa was identified as fibrinogen β chain by 2D-DIGE, this presumably resulting from the two different separative approaches. Content of β-actin was found higher in ‘TRG 3′ and ‘TRG 4′ T samples compared with other samples (Figure 4B), while, the content of β-tubulin was found higher only in association with the ‘TRG 4′ T sample (Figure 4B), accordantly with the observed 2D-DIGE data.

**Figure 5:**
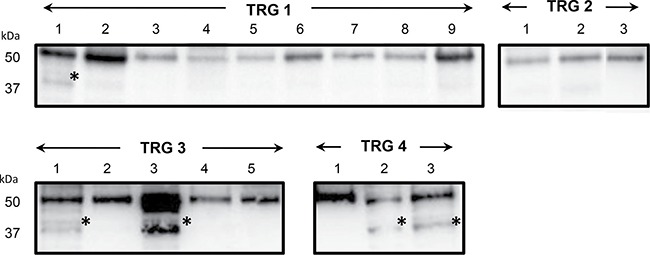
Immunoblotting validation of fibrinogen β chain expression in individual rectal cancer tissues of patients with good (TRG 1-2) or poor response (TRG 3 and TRG4) to neoadiuvant chemoradiotherapy Asterisk indicates the signal of a cross-reacting band at around 37 kDa.

Secondly, the increase in content of fibrinogen β chain fragment D in poor responsive patients was validated by immunoblotting in an independent cohort of 20 patients (Figure 5). Similarly to previous results, the fibrinogen β chain band of ~50 kDa increased in content in cancer tissues of ‘TRG 3′ and ‘TRG 4′ patients, as compared with ‘TRG 1–2′ ones. A higher content of the fibrinogen β chain band at ~37 kDa was observed in 1 to 12 patients of ‘TRG 1–2′ group (= 8%), in 2 to 6 patients of ‘TRG 3′ group (= 33%), and 2 to 3 patients of ‘TRG 4′ group (= 66%), thus confirming its higher content in poor responders. The presence of fibrinogen β chain in the gel portion at ~50 kDa and ~37 kDa was confirmed by MS analyses (data not shown).

### Protein interaction maps for biological processes

**Figure 6:**
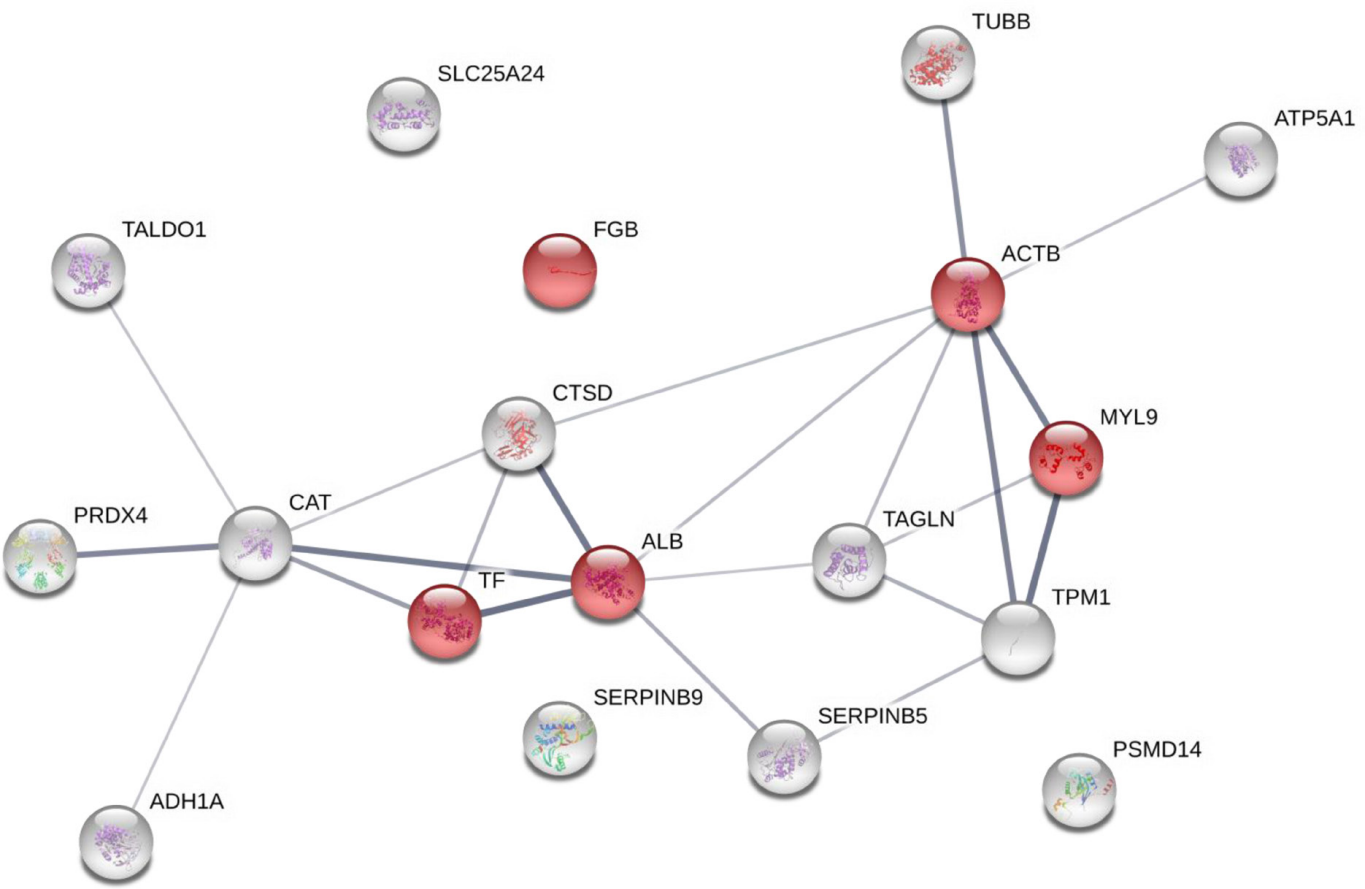
Protein-protein interaction maps of the overexpressed protein spots in rectal cancers of poor responders (TRG3 and TRG4) before therapy The interaction map of all the identified proteins increasing in content in poor responders is illustrated as confidence view, where the thickness of the connecting lines indicates the level of confidence. Stronger associations are represented by thicker lines. Each circle represents a protein. The proteins involved in ‘platelet activation’ and ‘blood coagulation’ are visualized in red. The STRING tool (http://string-db.org) was used to make the networks and analyse the biological processes.

Proteins overexpressed in good (‘TRG 1–2′) *versus* poor (‘TRG 3′ and ‘TRG 4′) responders were not found functionally connected (data not shown), this maybe coming from their different biological functions. While proteins increasing in content in poor responders were functionally connected (enrichment *p*-value: 8.94E-06), with the exception of three proteins (fibrinogen β chain, the 26S proteasome subunit and the mitochondrial carrier protein ScaMC-1 isoform 1). Their network was functionally enriched in two biological processes: ‘platelet aggregation’ (FDR: 0.0254) and ‘platelet activation’ (FDR: 0.0169, Figure 6, Supplementary Table 3). We focussed on proteins overexpressed in poor responders and built another network after including 10 proteins reported as candidate biomarkers responsive to neoadjuvant CRT in RC. In the resulting network (enrichment *p*-value: 8.8E-12) the protein VEGFA was added to proteins belonging to the ‘platelet activation’ biological process (FDR: 0.00115) (Supplementary Figure 2A), and the ‘negative regulation of apoptotic process’ (FDR: 0.000449) emerged as new interesting biological processes involving fibrinogen β chain (Supplementary Figure 2B and 2C).

## DISCUSSION

In this work, we identified a panel of predictive markers for nCRT response in LARC by using the 2D-DIGE quantitative proteomic approach.

We classified our patients for good or poor nCRT response referring to the TRG system according to Mandard histological classification based on the presence of residual cancer cells and fibrosis after therapy [24]. Patients with TRG values 1 and 2 were considered as good responders, while patients with TRG values 3 and 4 as poor responders to therapy.

Proteins were extracted from surgical biopsies collected before treatment from both cancer and the healthy normal tissues. A total of 30 spots were found to be differentially expressed in good *versus* poor responders, and corresponded to 27 unique protein identifications. In some cases, same proteins were detected from different spots, this coming from protein post-translational modifications or proteolytic cleavages, as reported by Rappsilber and Mann [25]. Moreover, some differential spots revealed more than one proteins, which it is possible to assume to have different relative amount, in agreement with Thiede *et al*. [26] findings of up to 5 proteins per spot.

Rectal tissues of poor responders (TRG 3 and TRG 4) were characterized before nCRT by a significant increase in content of the spots 471, 683 and 684, which were identified as ‘fibrinogen ß chain’ (spots 471), 3 ‘actin’ isoforms (spots 471, 683 and 684), ‘serpin B9′ (spot 471), ‘peroxiredoxin-4′ (spot 683), and 2 ‘cathepsin D’ isoforms (spots 683 and 684).

Composed of α, β, and γ polypeptides, fibrinogen is a principal factor in the maintenance of haemostasis, and it also displays vasoconstrictor, chemotactic and mitogen activities [27]. In malignancies, the presence of fibrinogen has been suggested to affect the progression of tumor cell growth and metastasis, as well as to influence adhesion, proliferation, and migration of tumor cells [28–30]. In LARC, pre-nCRT plasma fibrinogen level was found as predictive factor of complete response and disease recurrence following therapy [31]. Similarly, in another study pretherapeutic hyperfibrinogenemia was associated with a lower rate of tumor response to therapy and patient survival [32].

Because of its central role in coagulation and haemostasis, as well as its influence on tumor growth and metastasis, we focussed on fibrinogen β chain. We validated the increase in level of fibrinogen β chain at ~37 kDa in cancer tissues of the poor responders analysed by 2D-DIGE as well as in individual cancer tissues of an additional validation cohort of patients. We also found an increase in fibrinogen β chain at ~50 kDa in poor responders. The ~37 kDa band cross-reacting with fibrinogen β chain antibody was identified as the fibrinogen β chain portion between aminoacid positions 164 and 491, which is also known as fragment D (gi2781208; for crystal structure of fragment D refer to [33]). The coagulation cascade is known to culminate in the conversion of fibrinogen to fibrin by the protease thrombin, which cleaves fibrinopeptide B (31–44 aminoacid positions) from the the N-terminal part of fibrinogen β chain, the derived part of the protein having a mass of ~50 kDa [27]. Fibrin resulting in clot formation can be further degraded by proteases, the most efficient mainly being the plasmin, which cuts at various places including the 163–164 sites of fibrinogen β chain (cleavages sites described inhttp://www.uniprot.org/uniprot/P02675), thus originating a degraded fibrinogen β chain protein with a mass of ~37 kDa, the above mentioned fragment D. Interestingly, fibrinogen β chain fragment D was found by 2D analysis as cancer tissue-specific protein [34], and, even if this observation was found in gastric tumor, it may evidence the presence of common tumor-driven pathways involving fibrinogen. Accordantly to the coagulation/fibrinolysis processes, the observed increase in content of both the ~50 and ~37 kDa forms of fibrinogen β chain in cancer tissues of poor responders before nCRT may reflect an increase in the occurrence of some hemostatic processes specific of cancer microenvironment. Fibrinogen may be important for tissue repair or it may play a pathogenetic role in response to inflammatory processes.

Fibrinogen takes also part to the pathways of ‘platelet activation’ [35], that our network analyses showed as a significant biological process involving proteins overexpressed in poor responders. Platelet count has been described as an independent predictive factor for tumor response, high levels before therapy being associated with a poor response to nCRT [36–38]. All these data, including our proteins, may be suggestive of predictive platelet and blood coagulation pathways as negative predictors of RC responses to nCRT.

Together with the increased content in fibrinogen β chain and its fragment D, poor responders to nCRT also presented a high content of three actin isoforms (spots 471, 683 and 684). The assembly of actin into filaments is known to be dependent on several factors, including actin concentration, the presence of ATP and several actin-binding proteins [39]. An increase in the ATP synthase subunit α (spot 377) together with an increase in ATP synthase subunit β (spot 342) was detected together with actin in cancer tissues of poor responders. The involvement of actin cytoskeleton in tumorigenesis is well documented [40–41]. In patients of ‘TRG 4′ group, we also identified another protein involved in cell cytoskeleton: a tubulin β chain (spot 342). Moreover, cancer tissues of groups TRG 3 and, even not statistically significantly, TRG 4 had also a higher content of tropomyosin (spot 580), a core component of actin filaments influencing the mechanical properties of cells [42–43]. Globally, this higher increase in content of actin and related proteins in tumor tissues of poor responders may suggest a specific rearrangement of cell shape and motility.

Another highly abundant protein in poor responders was serpin B9 or proteinase inhibitor-9 (PI-9). This protein is known to protect tumor cells from apoptosis by binding and inactivating the cytotoxic granzyme B (GrB) molecules released by the immune-related cytotoxic cells [44]. This is in accord with our result of a higher PI-9 expression in tumor as compared with the healthy normal tissues. In spot 471, another serpin, serpin B5 or maspin, was identified, which was also present in the spot 492. In LARC patients with high level of apoptosis, the increase in staining of maspin correlated with a higher risk on local recurrence [45]. High levels of apoptosis index in RC biopsies before therapy have been previously described as predictors of a better response to nCRT [46].

Another protein overexpressed in poor responders, cathepsin D (CatD), has a regulatory role in apoptosis [47, 48]. This protein has been previously described in colorectal cancer as marker of poor prognosis associated with an increased metastastic risk [49–51]. Apoptosis plays a pivotal role in cancer scenario, so that it is also a popular target of many treatment strategies [52]. Our network analyses showed apoptosis as a significant biological process involving some proteins overexpressed in poor responders (i.g. fibrinogen β chain and serpinB9), together others coming from bibliography (p21, p53, CD44, VEGFA and EGFR). Several molecular markers related to regulation of cell cycle, apoptosis, or DNA repair have been proposed as candidate predictors of therapeutic response to CRT, but, to date, none has been definitively proven to be predictive of CRT response [10].

In pre-treatment cancer tissues of poor responders, we was also observed a high protein content for the peroxiredoxin-4 (Prx4). Peroxiredoxin-4 is an antioxidant enzyme playing a crucial role in inflammation, as well as promotion of cell proliferation and differentiation [53]. In colorectal cancer, Prx4 was overexpressed in cancer tissues, it correlated with the survival time of postoperative patients, and it was proposed as independent prognostic marker [54]. Overall these findings agree with our results, and may support the proposal of Prx4 as relevant predictive biomarker for treatment response.

In conclusion, this study highlighted the potential utility of a limited set of proteins as predictive tool for nCRT response in LARC. High levels of fragment D of fibrinogen β chain, actin, B9 and B5 serpins, cathepsin D and peroxiredoxin-4 were found associated with poor response to nCRT. Platelet adhesion/aggregation and apoptosis provide guidance for further investigation in RC responses to nCRT. The predictive value for fibrinogen β chain was confirmed in a validation cohort of patients. A complementary approach taking into account the role of tumor heterogeneity in nCRT response would be essential in the future to better decipher the potential molecular scenario predicting individual responses to therapy.

## MATERIALS AND METHODS

### Patients and tissues

Patient population consisted of 35 patients with LARC who received nCRT and then underwent surgical resection at the CRO National Cancer institute following ethical committee (CRO-2008-26 A.5) and informed consent (Table 1 and Supplementary Table 2). Eligibility criteria of patients were the following: histologically confirmed diagnosis of primary resectable LARC, confirmed absence of distant metastasis, age ≥ 18 years, and Caucasian ethnicity. The nCRT was based on fluoropyrimidines (either 5FU or capacetabine) with or without oxaliplatin combined with a dose of 50.4 Gy or 55.0 Gy of radiation. All patients had biopsy-proven adenocarcinoma of the rectum (1–10 cm from the pectinate line). The study population comprised patients with a median or distal tumor region accordantly to the criteria for neoadiuvant therapy [22]. These patients were enrolled for proteomic investigations from a registry, according to the above mentioned eligibility criteria. All patients received rectal tumor biopsy before treatment and had histological confirmation.

Biopsies were collected from rectal cancer (T) and healthy normal tissue (N), which was taken above 5 cm from the tumor margin and histologically confirmed as normal.

All biopsies were handled in an identical way. After collection, biopsies were immediately put on ice and then stored at −80°C before protein extraction. Fifteen T and N pairs were used for two dimensional difference in gel electrophoresis (2D-DIGE) (Table 1), while 19 T samples were examined as independent validation set by western blot (WB) (Supplementary Table 2). Characteristics of patients and tumor samples are given in Table 1 and Supplementary Table 2.

### Histological evaluation

Pathological tumor staging of the resected specimen was performed in accordance with the guidelines of the American joint committee of cancer. Treatment efficacy defined as TRG was assessed after surgery on surgical specimens through serial histological examinations of whole tumor area, together with sital, proximal and radial resection margins, accordingly to a 5-tier scoring system: ‘TRG 1′, complete regression; ‘TRG 2′, major regression, rare neoplastic cells in prevalent fibrosis; ‘TRG 3′, moderate regression, fibrosis >50%; ‘TRG 4′, partial regression, fribrosis < 50%, and ‘TRG 5′, no regression [24].

Patients with a TRG 1 or 2 were considered as ‘good’ responders, while patients with TRG 3 and 4 and having a ‘moderate’ and ‘partial’ response to nCRT were considered as ‘poor responders’. All patients were followed-up every 3 months for the first 2 years, and every 6 months thereafter up to 5 years.

### Two dimensional difference in gel electrophoresis (2D-DIGE)

The frozen biopsies were homogenated with sample grinding kit (GE Healthcare, Uppsala, Sweden) in cold lysis buffer (4% (w/v) CHAPS, 7 M Urea, 2 M Thiourea, 30 mM Tris, pH 8.5) containing 100 mM DTT and a protease inhibitor cocktail (Sigma-Aldrich, St. Louis, MO, USA). Proteins were then treated with 2D Clean Up kit (GE Healthcare) to improve the quality of 2D gels, and resuspended in rehydration buffer for 2D analysis (7 M Urea, 2 M Thiourea, 4% (w/v) CHAPS, 0.5% (v/v) IPG buffer 3-10 NL) and quantified with the Bradford-based assay (Bio-Rad, Milan, Italy), as previously reported by *Repetto et al.* [55]. For 2D-DIGE minimal labeling, the protein extracts were labeled with cyanine dyes (CyDyes) according to the manufacturer's protocol (CyDye DIGE Fluor minimal dyes; GE Healthcare). The entire project consisted of 15 gels, each gel containing 2 protein extracts (25 μg) from biopsies of RC-affected (T) and biopsies of the adjacent control tissues (N) of the same patient, respectively, each labeled with Cy3 or Cy5 together with the internal standard (Cy2-labeled; 25 μg). The Cy2-labeled pool used in 2D-DIGE is representative of the all samples analysed because it is formed from equal amounts of each protein sample in the experiment; it is usefull to reduce inter-gel variation and accurately quantitate spots, and it provides a consistent spot map on all gels in an experiment, thus facilitating spot matching. Proteins were firstly separated by isoelectrofocusing (IEF) on 3-10 NL pH gradient dry strips (IPG, Bio-Rad) and then on 8–16 % Criterion TGX precast midi gels (Bio-Rad). For preparative gels, 400 μg of unlabelled proteins pooled from equal amounts of all samples were separated, and stained with Coomassie Brilliant Blue CBB G-250 and ammonium sulfate. After gel scanning (Typhoon Trio 940™ laser scanner; GE Healthcare), 2D-differential analysis (DeCyder software version 6.5, GE Healthcare) was performed. First, T-tissue proteome maps of patients categorized as either ‘TRG 3′ or ‘TRG 4′ were compared with those reported a ‘TRG 1–2′ (Table 2). Secondly, we compared the protein expression of the T-tissues with that of the N-tissues of the same patients. Gel image pairs were processed by the Decyder Differential In-gel Analysis (DIA) module to co-detect and differentially quantify the protein spots in the images; the internal standard sample was used as a reference to normalize the data, so the rest of the normalized spot maps could be compared among them. The Decyder Extended analysis (EDA) module was used for multivariate analysis of protein expression data, derived from Biological variation analysis (BVA) module, which performs a gel-to-gel matching of the internal standard spot maps from each gel. Student's *t* test and Principal component analysis (PCA) were performed to test the statistical significance of the differential proteins among the three TRG groups. Spots for which relative (spot) volume changed at least 1.5-fold (increase or decrease) were considered to be altered in abundance at 95% confidence level (Student's *t*-test; *p* < 0.05). The 1.5-fold difference value is setted as default by the software Decyder as the minimal value to consider spot content variation (increase or decrease) as consistent, and it is significant if *p*-value is < 0.05. These proteins have been picked from the preparative gel as described below.

### Protein identification by mass spectrometry (MS)

Spots protein of interest were excised from the preparative gel with Screen Picker (Proteomics Consult, Kampenhout, Belgium), and peptides were extracted with trifluoroacetic acid after destained and trypsin-digestion [55]. Resulting peptides were analysed by mass spectrometry (MS) using either an LC/MSD XCT Ultra Ion Trap equipped with a 1100 HPLC system and a chip cube (Agilent Technologies, Santa Clara, California, USA) or a LTQ XL-Orbitrap ETD equipped with a HPLC NanoEasy-PROXEON (Thermo Fisher Scientific, Waltham, Massachusetts, USA). Database searches were done with the MASCOT search engine version 2.3 against SwissProt and NCBInr (Matrix Science, London, UK). In those spots resulting to contain more then one proteins, possible post-translational phosphorylations were searched in STY aminoacids. For each identified protein, subcellular location was controlled in UniProtKB as Gene Ontology (GO) annotation (http://www.uniprot.org/uniprot).

### Immunoblotting

The differential abundance of some differentially expressed proteins of interest was validated by immunoblot analyses. A first validation was performed on 3 pools of proteins, using all 15 paired samples analysed by 2D-DIGE. A second validation was performed on individual proteins extracted from cancer tissues of an additional cohort of 20 patients. Ten μg of proteins were fractionated on 12% Criterion TGX Stain-Free gels and, after gel image acquisition with the Chemidoc system (BIO-RAD) and electrotransfer onto nitrocellulose membranes. Membranes were incubated with the monoclonal antibodies anti-fibrinogen β chain [1F9] (1:500; GeneTex) and anti-β-actin (1:1000, Abcam), and the polyclonal anti-β-tubulin (1:3000, Santa-Cruz). Antibody-bound proteins were detected by enhanced chemiluminescence using the Chemidoc system after incubation with ECL HRP-conjugated secondary antibodies (1:25000 dilution, GE Heathcare) and reaction with ECL Prime Western Blotting detection reagent (GE Healthcare). The image of the gel acquired before its transfer was used as control for equal protein loading among samples.

### Protein network analyses

Biological processes of the identified proteins of interest were analysed using a dedicated tool of STRING (version 9.1;http://string-db.org) based on GO annotations [56]. In order to better understand the possible role of the differential proteins in cancer tissues before therapy, we integrated our pathway data with those from other proteins, which are reported as candidate gene biomarkers responsive to neoadjuvant CRT in LARC [11]. In particular, we included to our analyses the following proteins: (1) cyclin D1 (CCND1, regulatory subunit of cyclin-dependent kinases CDK4 and CDK6, the D1-CDK4 complex promoting passage through the G1 phase); (2) epidermal growth factor receptor (EGFR, whose constant activation if mutated produces uncontrolled cell division); (3) antigen KI-67 (MKI67, involved in cell cycle regulation and cellular marker for proliferation); (4) thymidilate synthase (TYMS, playing a crucial role in the early stages of DNA biosynthesis); (5) cyclin-dependent kinase inhibitor 1A (p21/CDKN1A, by which p53/TP53 mediates its role as an inhibitor of cellular proliferation in response to DNA damage); (6) tumor protein p53 (TP53, involved in cell cycle regulation and growth arrest or apoptosis); (7) vascular epidermal growth factor (VEGFA, a signal protein produced by cells that stimulates vasculogenesis and angiogenesis); (8) mutL homolog 1 (MLH1, component of the post-replicative DNA mismatch repair system); (9) carcinoembryonic antigen (CEA, involved in cell adhesion); (10) CD44 antigen (CD44, involved in many physiological activities such as cell proliferation, differentiation, migration and angiogenesis). As interaction tool we adopted the STRING evidence view, which suggests a functional link among genes in the following cases: neighborhood in the genome, gene fusions, cooccurrence accross genomes, co-expression, exprimental/biochemical data, association in curated databases, co-mentioned in PubMed abstracts.

## SUPPLEMENTARY MATERIALS FIGURES AND TABLES




