# Supplementary material for: Identification of protein clusters predictive of tumor response in rectal cancer patients receiving neoadjuvant chemo-radiotherapy
Source: Oncotarget. 2017 Mar 9;8(17):28328–41. doi: 10.18632/oncotarget.16053 (PMC5438653; doi:10.18632/oncotarget.16053)
Supplement: Supplementary file 1 [file oncotarget-08-28328-s001.pdf]

Identification of protein clusters predictive of tumor response in rectal cancer patients receiving neoadjuvant chemo-radiotherapy

Supplementary Materials

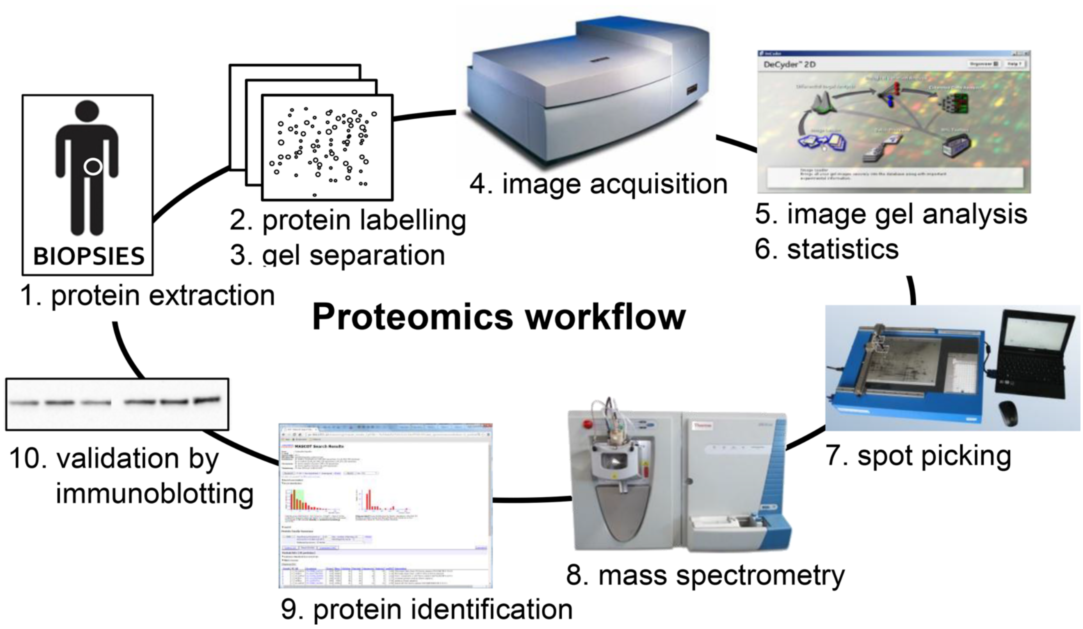

Supplementary Figure 1: A schematic illustration of the proteomic workflow adopted. Proteins have been extracted from both rectal cancer (T) and the healthy normal tissue (N) biopsies of 15 patients (1). After labelling (2), proteins were separated by first and second dimension (3). Gel images were acquired (4) and analysed with a dedicated software (5), which allowed to detect and statistically analyse the differentially expressed spots in 'TRG 1-2' group *versus* either 'TRG 3' or 'TRG4'. These spots of interest were excised from a preparative gel (7), digested with trypsin and separated by mass spectrometry (8). For each spot, MS/MS spectra were searched against sequence database to get protein identification(s) (9). The differential expression of some identified proteins of interest was validated by immunoblotting (10). Details are explained in Methods section.

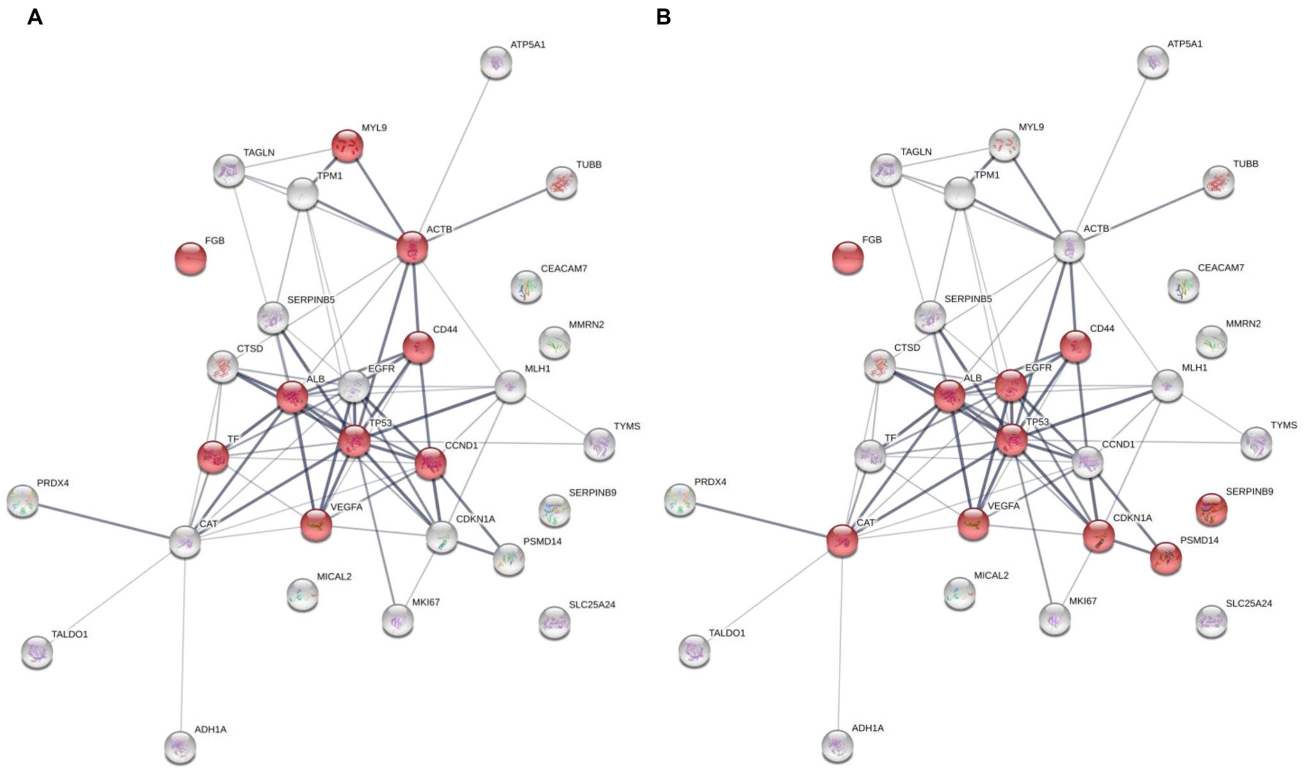

Supplementary Figure 2: Protein-protein interaction maps of the overexpressed protein spots in rectal cancers of poor responders (TRG3 and TRG4) before therapy together with some key proteins known to be candidate biomarkers responsive to neoadjuvant CRT. The interaction map is illustrated as confidence view, where the thickness of the connecting lines indicates the level of confidence. Stronger associations are represented by thicker lines. Each circle represents a protein. Panels visualize in red proteins involved in 'platelet activation' (**A**) and 'negative regulation of apoptosis' (**B**). The STRING tool (http://string-db.org) was used to make the networks and analyse the biological processes.

Supplementary Table 1: Clinicopathological characteristics of patients affected by rectal cancer of median and distal localization, accordantly to the criteria for the nCRT [21]

Patient nr.	Sex	Age	Pre-CRT stage TNM	TRG	TRG group of analysis
1	M	74	T3	1	1-2
2	M	78	T2N0	1	
3	M	61	T3N0	1	
4	F	60	T3N0	1	
5	M	64	T3N+	1	
6	M	55	T3N0	1	
7	M	63	T3N+	1	
8	M	49	T3N+	1	
9	F	57	T3N+	1	
10	F	60	T3N0	2	
11	M	73	T3N+	2	
12	F	75	T3N0	2	
1	F	49	T3/4N+	3	3
2	M	70	T3N0	3	
3	F	78	T3N0	3	
4	M	43	T2N0	3	
5	F	65	T3N0	3	
1	M	78	T3N+	4	4
2	F	77	T3N+	4	
3	M	48	T3N+	4	

Pathological responses were evaluated with the tumor regression grade (TRG) system. Their biopsies were used as independent validation set for fibrinogen beta protein, as described in 'Material and methods' section.

Supplementary Table 2: Identification of differentially expressed spots rectal cancer by mass spectrometry. See Supplementary_Table_2

Supplementary Table 3: List of 9 biological processes (*p*-value_fdr < 0.05) related to all the differential proteins (both up- and down-regulated) found in tumor regression groups 'TRG 1-2' *versus* 'TRG 3' or 'TRG 4' of rectal cancer

GO_id	Term	Number of proteins	*p*-value_fdr
GO:0030168	platelet activation	6	3.58E-03
GO:0002576	platelet degranulation	4	2.27E-02
GO:0042060	wound healing	7	3.55E-02
GO:0070527	platelet aggregation	3	3.55E-02
GO:0009611	response to wounding	7	4.32E-02
GO:0007596	blood coagulation	6	4.70E-02
GO:0050817	coagulation	6	4.70E-02
GO:0007599	hemostasis	6	4.70E-02
GO:0001775	cell activation	6	9.09E-02

Biological processes were analysed with STRING (GO, Gene Ontology).
