# Supplementary material for: Identification of protein clusters predictive of tumor response in rectal cancer patients receiving neoadjuvant chemo-radiotherapy
Source: Oncotarget. 2017 Mar 9;8(17):28328–41. doi: 10.18632/oncotarget.16053 (PMC5438653; doi:10.18632/oncotarget.16053)
Supplement: Supplementary file 2 [file oncotarget-08-28328-s002.doc]

**Supplementary Table 2.** Identification of differentially expressed spots rectal cancer by mass spectrometry.

| **Class ^a)^** | **Spot nr. ^b)^** | **Database** | **Accession** | **Protein annotation** | **Score/**  **Seq. coverage %** | **Matches/ Sequences** | **Nr peptides with ion score >** | **MS Instrument** |
| --- | --- | --- | --- | --- | --- | --- | --- | --- |
| **a)** | 114 | SwissProt | [MVP_HUMAN](http://pc-8011353-12/mascot/cgi/protein_view.pl?file=..%2Fdata%2F20151123%2FF001908.dat;_msresflags=3138;_msresflags2=10;ave_thresh=28;db_idx=2;hit=MVP_HUMAN;px=1;report=20) | **Major vault protein** | 327/20 | 37/21 | 14 | Orbitrap |
|  | 150 | SwissProt | [IMMT_HUMAN](http://pc-8011353-12/mascot/cgi/protein_view.pl?file=..%2Fdata%2F20151221%2FF001965.dat;_msresflags=3138;_msresflags2=10;ave_thresh=28;db_idx=2;hit=IMMT_HUMAN;px=1;report=20) | **Mitochondrial inner membrane protein or mitofilin** | 708/31 | 52/29 | 19 | Orbitrap |
|  | 152 | NCBInr | [gi|154354966](http://pc-8011353-12/mascot/cgi/protein_view.pl?file=..%2Fdata%2F20151123%2FF001905.dat;_msresflags=3138;_msresflags2=10;ave_thresh=28;db_idx=1;hit=gi|154354966;px=1;report=20) | **Mitochondrial inner membrane protein isoform 3 or mitofilin** | 509/39 | 54/29 | 15 | Orbitrap |
|  | 333 | NCBInr | [gi|220702506](http://pc-8011353-12/mascot/cgi/protein_view.pl?file=..%2Fdata%2F20151123%2FF001906.dat;_msresflags=3138;_msresflags2=10;ave_thresh=29;db_idx=1;hit=gi|220702506;px=1;report=20) | **Chain A, TapasinERP57 HETERODIMER** | 2271/49 | 355 /28 | 21 | Orbitrap |
|  | 358 | SwissProt | [TCPB_HUMAN](http://pc-8011353-12/mascot/cgi/protein_view.pl?file=..%2Fdata%2F20151123%2FF001907.dat;_msresflags=3138;_msresflags2=10;ave_thresh=28;db_idx=2;hit=TCPB_HUMAN;px=1;report=20) | **T-complex protein 1 subunit ß** | 996/36 | 62/20 | 16 | Orbitrap |
| **b)** | 908 | SwissProt | PPIA_HUMAN | **Peptidyl-prolyl cis-trans isomerase or Cyclophilin A** | 513/29 | 82/6 | 5 | Orbitrap |
|  | 289 | NCBInr | [gi|34228](http://pc-8011353-12/mascot/cgi/protein_view.pl?file=..%2Fdata%2F20150810%2FF001846.dat;_msresflags=3138;_msresflags2=10;ave_thresh=44;db_idx=1;hit=gi|34228;px=1;report=20) | **Unnamed protein product, putative lamin A precursor** | 663 /36 | 52/25 | 12 | Ultra Ion Trap |
|  | 571 | NCBInr | [gi|1703319](http://pc-8011353-12/mascot/cgi/protein_view.pl?file=..%2Fdata%2F20150810%2FF001840.dat;_msresflags=3138;_msresflags2=10;ave_thresh=43;db_idx=1;hit=gi|1703319;px=1) | **Annexin A4** | 851/45 | 43/14 | 12 | Ultra Ion Trap |
| **c)** | 373 | NCBInr | gi|148491091 | **Calcium-binding mitochondrial carrier protein SCaMC-1 isoform 1** | 200/30 | 17/12 | 4 | Ultra Ion Trap |
|  | 535 | SwissProt | AK1A1_HUMAN | **Alcohol dehydrogenase [NADP(+)]** | 704/35 | 84/13 | 9 | Orbitrap |
|  | 811 | SwissProt | [MYL9_HUMAN](http://pc-8011353-12/mascot/cgi/protein_view.pl?file=..%2Fdata%2F20151123%2FF001909.dat;_msresflags=3138;_msresflags2=10;ave_thresh=28;db_idx=2;hit=MYL9_HUMAN;px=1;report=20) | **Myosin regulatory light polypeptide 9** | 203 /29 | 13/5 | 5 | Orbitrap |
|  | 377 | SwissProt | [ATPA_HUMAN](http://pc-8011353-12/mascot/cgi/protein_view.pl?file=..%2Fdata%2F20151123%2FF001912.dat;_msresflags=3138;_msresflags2=10;ave_thresh=29;db_idx=2;hit=ATPA_HUMAN;px=1;report=20) | **ATP synthase subunit α, mitochondrial** | 1135/29 | 123/17 | 8 | Orbitrap |
|  | 471 | SwissProt, NCBInr | FIBB_HUMAN | **Fibrinogen ß chain** | 608/26 | 42/13 | 9 | Orbitrap |
|  |  | SwissProt | ACTB_HUMAN | **Actin, cytoplasmic 1** | 460/32 | 32/10 | 9 | Orbitrap |
|  |  | SwissProt | SPB9_HUMAN | **Serpin B9** | 431/33 | 21/11 | 11 | Orbitrap |
|  |  | SwissProt | SPB5_HUMAN | **Serpin B5** | 204/32 | 11/9 | 9 |  |
|  | 580 | NCBInr | [gi|63252900](http://pc-8011353-12/mascot/cgi/protein_view.pl?file=..%2Fdata%2F20151123%2FF001918.dat;_msresflags=3138;_msresflags2=10;ave_thresh=29;db_idx=1;hit=gi|63252900;px=1;report=20) | **Tropomyosin α-1 chain isoform 4** | 670/53 | 40/22 | 17 | Orbitrap |
|  | 284 | SwissProt | [CATA_HUMAN](http://pc-8011353-12/mascot/cgi/protein_view.pl?file=..%2Fdata%2F20151123%2FF001911.dat;_msresflags=3138;_msresflags2=10;ave_thresh=27;db_idx=2;hit=CATA_HUMAN;px=1;report=20) | **Catalase** | 451/24 | 30/14 | 8 | Orbitrap |
|  | 573 | SwissProt | [TALDO_HUMAN](http://pc-8011353-12/mascot/cgi/protein_view.pl?file=..%2Fdata%2F20151123%2FF001917.dat;_msresflags=3138;_msresflags2=10;ave_thresh=28;db_idx=2;hit=TALDO_HUMAN;px=1;report=20) | **Transaldolase** | 86/12 | 5/5 | 4 |  |
|  | 492 | SwissProt | FIBB_HUMAN | **Fibrinogen ß chain** | 500/26 | 25/13 | 8 | Orbitrap |
|  |  | SwissProt | ACTB_HUMAN | **Actin, cytoplasmic 1** | 336/25 | 16/8 | 6 |  |
|  |  | NCBInr | gi|60817455 | **Serpin B5** | 138/27 | 15/11 | 5 |  |
|  | 683 | SwissProt | ACTB_HUMAN | **Actin, cytoplasmic 1** | 382/22 | 27/7 | 6 | Orbitrap |
|  |  | SwissProt | PRDX4_HUMAN | **Peroxiredoxin-4** | 290/28 | 17/8 | 6 |  |
|  |  | SwissProt | CATD_HUMAN | **Cathepsin D** | 243/19 | 15/8 | 3 |  |
|  | 684 | SwissProt | ACTB_HUMAN | **Actin, cytoplasmic 1** | 1086/40 | 135/12 | 9 | Orbitrap |
|  |  | SwissProt | CATD_HUMAN | **Cathepsin D** | 243/19 | 15/8 | 6 | Orbitrap |
| **d)** | 193 | SwissProt | LMNA_HUMAN | **Prelamin-A/C** | 646/49 | 55/32 | 16 | Ultra Ion Trap |
|  |  | SwissProt | TRFE_HUMAN | **Serotransferrin** | 518/21 | 36/16 | 10 | Ultra Ion Trap |
|  | 342 | SwissProt | ATPB_HUMAN | **ATP synthase subunit ß, mitochondrial** | 980/44 | 48/18 | 16 | Ultra Ion Trap |
|  |  | SwissProt | TBB5_HUMAN | **Tubulin ß chain** | 917/45 | 51/19 | 16 | Ultra Ion Trap |
|  | 264 | NCBInr | [gi|157830361](http://pc-8011353-12/mascot/cgi/protein_view.pl?file=..%2Fdata%2F20151123%2FF001910.dat;_msresflags=3138;_msresflags2=10;ave_thresh=28;db_idx=1;hit=gi|157830361;px=1;report=20) | **Chain A, Human Serum Albumin In A Complex With Myristic Acid And Tri- Iodobenzoic Acid** | 1099/32 | 199/25 | 17 | Orbitrap |
|  | 471 | SwissProt, NCBInr | FIBB_HUMAN | **Fibrinogen ß chain** | 608/26 | 42/13 | 9 | Orbitrap |
|  |  | SwissProt | ACTB_HUMAN | **Actin, cytoplasmic 1** | 460/32 | 32/10 | 9 | Orbitrap |
|  |  | SwissProt | SPB9_HUMAN | **Serpin B9** | 431/33 | 21/11 | 11 | Orbitrap |
|  | 683 | SwissProt | ACTB_HUMAN | **Actin, cytoplasmic 1** | 382/22 | 33/7 |  | Orbitrap |
|  |  | SwissProt | PRDX4_HUMAN | **Peroxiredoxin-4** | 290/28 | 17/8 | 6 | Orbitrap |
|  |  | SwissProt | CATD_HUMAN | **Cathepsin D** | 243/19 | 15/8 | 3 | Orbitrap |
|  | 425 | SwissProt | [ACTBM_HUMAN](http://pc-8011353-12/mascot/cgi/protein_view.pl?file=..%2Fdata%2F20151123%2FF001913.dat;_msresflags=3138;_msresflags2=10;ave_thresh=29;db_idx=2;hit=ACTBM_HUMAN;px=1;report=20) | **Putative ß-actin-like protein 3 (POTE ankyrin domain family member K)** | 496/3 | 50/1 | 1 | Orbitrap |
|  | 741 | NCBInr | [gi|194173391](http://pc-8011353-12/mascot/cgi/protein_view.pl?file=..%2Fdata%2F20151123%2FF001923.dat;_msresflags=3138;_msresflags2=10;ave_thresh=29;db_idx=1;hit=gi|194173391;px=1;report=20) | **Immunoglobulin light chain** | 1007/40 | 65/6 | 5 | Orbitrap |
|  | 553 | SwissProt | PSDE_HUMAN | **26S proteasome non-ATPase regulatory subunit 14** | 86/12 | 5/5 | 3 | Orbitrap |
|  | 684 | SwissProt | ACTB_HUMAN | **Actin, cytoplasmic 1** | 1086/40 | 135/12 | 9 | Orbitrap |
|  |  | SwissProt | CATD_HUMAN | **Cathepsin D** | 243/19 | 15/8 | 6 | Orbitrap |
|  | 377 | SwissProt | [ATPA_HUMAN](http://pc-8011353-12/mascot/cgi/protein_view.pl?file=..%2Fdata%2F20151123%2FF001912.dat;_msresflags=3138;_msresflags2=10;ave_thresh=29;db_idx=2;hit=ATPA_HUMAN;px=1;report=20) | **ATP synthase subunit α, mitochondrial** | 1135/29 | 123/17 | 8 | Orbitrap |
|  | 828 | NCBInr | [gi|62897565](http://pc-8011353-12/mascot/cgi/protein_view.pl?file=..%2Fdata%2F20151123%2FF001924.dat;_msresflags=3138;_msresflags2=10;ave_thresh=28;db_idx=1;hit=gi|62897565;px=1;report=20) | **Transgelin variant** | 564/61 | 43/12 | 10 | Orbitrap |

^a)^Classes: a, up-regulated spots in 'TRG 1-2' *versus* 'TRG 3'; b, up-regulated spots in 'TRG 1-2' *versus* 'TRG 4'; c, up-regulated spots in 'TRG 3' *versus* 'TRG 1-2'; d, up-regulated spots in 'TRG 4' *versus* 'TRG 1-2'; ^b)^spot nr., spot numbers refer to Figure 2.
